# Bacterial Adhesion and Biofilm Formation on Textured Breast Implant Shell Materials

**DOI:** 10.1007/s00266-018-1234-7

**Published:** 2018-10-01

**Authors:** Garth A. James, Laura Boegli, John Hancock, Lisa Bowersock, Albert Parker, Brian M. Kinney

**Affiliations:** 1 Center for Biofilm Engineering, Montana State University, 366 Barnard Hall, Bozeman, MT 59717 USA; 2 Establishment Labs, Santa Barbara, CA USA; 3 Keck School of Medicine, Division of Plastic Surgery, University of Southern California, Beverly Hills, CA 90212 USA

**Keywords:** Bacteria, Attachment, Biofilm, Breast implant, Silicone, Texture, Contracture, ALCL

## Abstract

**Background:**

Bacterial biofilms have been implicated with breast implant complications including capsular contracture and anaplastic large-cell lymphoma. The actual mechanisms for either are still under active investigation and are not clear. Due to their increased surface area, implants with textured surfaces may harbor greater biofilm loads than those with smooth surfaces.

**Methods:**

Biofilm formation on the outer surface material was compared using implants with various surface areas and roughness, including Natrelle^®^ (Smooth), SmoothSilk^®^/SilkSurface^®^ (Silk), VelvetSurface ^®^ (Velvet), Siltex^®^, and Biocell^®^. The roughness and surface area of each material were assessed using non-contact profilometry. Bacterial attachment (2 h) and biofilm formation (24 h) were evaluated for *Staphylococcus epidermidis*, *Pseudomonas aeruginosa*, and *Ralstonia pickettii* over nine independent experiments using a CDC biofilm reactor and viable plate counts (VPCs) as well as confocal scanning laser microscopy. VPCs of the textured implants were compared relative to the Smooth implant.

**Results:**

Surface areas increased with roughness and were similar among the three least rough implants (Smooth, Silk, and Velvet) and among the roughest implants (Siltex and Biocell). Overall, VPC indicated there was significantly more bacterial attachment and biofilm formation on the Siltex and Biocell implants than the Silk or Velvet implants, although there were differences between species and time points. CSLM confirmed the formation of thicker biofilms on the implants with rougher surface textures.

**Conclusion:**

This in vitro study confirmed that implant surfaces with rougher texture, resulting in more surface area, harbored greater biofilm loads than those with smoother surfaces.

**No Level Assigned:**

This journal requires that authors assign a level of evidence to each submission to which Evidence-Based Medicine rankings are applicable. This excludes Review Articles, Book Reviews, and manuscripts that concern Basic Science, Animal Studies, Cadaver Studies, and Experimental Studies. For a full description of these Evidence-Based Medicine ratings, please refer to the Table of Contents or the online Instructions to Authors www.springer.com/00266.

## Introduction

Bacterial biofilms have been implicated with breast implant complications including capsular contracture [1–4], double-capsule formation [5], and breast implant-associated anaplastic large-cell lymphoma (BI-ALCL) [6]. The reduction of capsular contracture and ALCL using steps to reduce the introduction of bacteria during surgery provided indirect evidence of the role of bacteria in these conditions [7]. Due to their increased surface area, implants with highly textured surfaces may harbor greater biofilm loads than those with smooth surfaces.

Gram-positive bacteria such as *Staphylococcus epidermidis* and other coagulase-negative staphylococci, along with *Cutibacterium* (formerly *Propionibacterium*), are the most commonly isolated bacteria from capsular contracture [1–4]. In a recent study of BI-ALCL, Gram-negative bacteria including pseudomonads and the genus *Ralstonia* in particular were the most commonly detected [8].

In this study, bacterial attachment and biofilm formation on the outer surface material of breast implants by *S. epidermidis*, *Pseudomonas aeruginosa,* and *Ralstonia pickettii* were compared using implants with various surface areas and roughness. The implants included Natrelle^®^ (Smooth), SmoothSilk^®^/SilkSurface^®^ (Silk), VelvetSurface ^®^ (Velvet), Siltex^®^, and Biocell^®^. No Mentor or Motiva smooth implant surfaces were tested, as the surface on scanning electron microscopy showed no gross differences than Natrelle^®^ (Smooth) on direct observation. The textured implants in each experiment were compared after normalizing bacterial responses to the smooth implant response in the same experiment. Overall, the results indicated that rougher textures (Siltex and Biocell) with more surface area had more bacterial attachment and biofilm formation than those with smoother textures and less surface area (Silk and Velvet). Interestingly, there were differences between species and time points. For *P. aeruginosa,* there were significant differences between textures for bacterial attachment but not for biofilm formation. In contrast, *S. epidermidis* and *R. pickettii* had more significant differences in biofilm formation than bacterial attachment between textures.

## Materials and Methods

### Test Materials

Four silicone surfaces and one untextured control material were compared (descriptions are shown in Table 1). Sterile implants were cut on the equator side and the gel extracted. For surface metrology, 2-cm^2^ shell sections were cut from the shells with a clean scalpel. For bacterial attachment and biofilm formation assays, 1.0-cm disks were cut with a rotary punch, using ethanol as a lubricant. The disks were then attached to 1.27-cm-diameter polycarbonate CDC Biofilm Reactor (CDC-BR) sample coupons (see below) using medical-grade silicone adhesive (A-100, Factor II, Inc.) to provide a more rigid support material for insertion into the CDC-BR coupon holders. The adhesive was cured for at least 24 h at room temperature.

**Table 1:** Breast implant types included in this study

Designation	Trade name	Manufacturer	Manufacturing method
Smooth	Natrelle^®^	Allergan	Dipping
Silk	SmoothSilk^®^/SilkSurface^®^	Motiva	Negative imprint
Velvet	VelvetSurface^®^	Motiva	Negative imprint
Biocell	Biocell^®^	Allergan	Salt loss
Siltex	Siltex^®^	Mentor	Imprint

### Surface Metrology

Surface area and roughness were measured over an area of 4.8 mm^2^ ± 0.2 mm^2^ with two replicates of the apex, base, and equator of each implant type using a non-contact profilometer, µSurf Mobile, in accordance with ISO 25178-2:2012 Surface texture: Areal-Terms, definitions, and surface texture parameters. The profilometry testing for the Siltex and Biocell textures was completed using a 20× imaging lens. The testing for the Silk, Velvet, and Smooth surfaces was completed using a 50× imaging lens. All parameters were calculated using µSoft Analysis extended software package version 7.3.7835.

### Bacteria

Three tests were performed using each of *Staphylococcus epidermidis* ATCC 35984, *Pseudomonas aeruginosa* ATCC 9027, and *Ralstonia pickettii* ATCC 27511 for a total of nine independent tests. These strains are maintained at − 70 °C as frozen stock cultures. Overnight cultures were prepared in Tryptic Soy Broth (TSB), one day prior to each experimental run.

### Biofilm Test System

Bacterial attachment and biofilm formation were assessed using a CDC Biofilm Reactor (CDC-BR, model CBR 90, Biosurface Technologies Corporation, Bozeman, MT) developed by the Centers for Disease Control and used to study biofilms formed by various bacterial species. A protocol using this reactor has been approved by ASTM as a standard method for growing repeatable *P. aeruginosa* biofilms on polycarbonate surfaces (Designation E 2562-17). The testing described herein used a modified protocol to enable the evaluation of bacterial attachment and biofilm formation on breast implant outer shell samples. The CDC-BR consists of a 1-l vessel with eight polypropylene coupon holders. Each coupon holder or rod can accommodate three 1.27-cm-diameter sample coupons, suspended from the lid. Liquid growth medium enters through the top of the vessel and exits via a side-arm discharge port. A magnetic stir bar incorporating a mixing blade provides fluid mixing and surface shear.

The disk/coupon assemblies were inserted into CDC-BR sample rods, and these were inserted into a CDC-BR lid. The disk/coupon/rod assemblies were soaked for 10 min with agitation in an enzymatic detergent, then rinsed six times with 1 l of deionized water each, and then autoclaved at 20 lb per square inch of pressure and a temperature of 123 °C for 35 min. Prior to use, the samples were conditioned by incubating them for 30 min at 37 °C in sterile de-complemented human serum diluted 1–10 in phosphate-buffered saline (PBS). After incubation, the samples were rinsed with sterile saline.

A CDC-BR (without coupon holders) containing approximately 400 ml of growth medium (10%-strength brain–heart infusion broth with 0.5% adult bovine serum) was inoculated with an overnight culture of the test species to provide an initial bacterial density of approximately 10^6^ colony-forming units per milliliter (CFU/ml), and the CDC-BR lid assembly, with the conditioned samples, was inserted. The CDC-BR was then operated with continuous flow (2.7 ml/min) and stirring (125 rpm) at 37 °C.

### CDC-BR Sampling

Bacterial attachment was assessed after 2 h of incubation. This time period was too short for significant biofilm growth to occur on the surfaces. Four sample rods were removed from the CDC-BR and rinsed to remove planktonic and loosely adhered bacteria by dipping the coupon holder into two consecutive beakers containing sterile phosphate-buffered saline (PBS). The removed sample rods were replaced with “dummy” sample rods that did not contain samples. Biofilm formation was assessed after 24 h of incubation. Disk/coupon assemblies were collected as described above for the bacterial attachment assessment.

In each of the three repeat experiments for each microbial species, two samples of each material type were analyzed by plate count and 3–4 samples of selected material types were analyzed by confocal scanning laser microscopy (CSLM). The 3–4 material types for CSLM were selected so that all material types at each time point were analyzed over the course of the three experiments. Samples of the CDC-BR bulk fluid were also collected at the bacterial attachment (2 h) and biofilm formation (24 h) time points.

### Plate Count Analysis

Breast implant material samples (1.0-cm disks) were removed from the polycarbonate coupons with a sterile blade and placed in vials containing 10 ml of sterile PBS. Bulk fluid samples (1 ml) were placed in vials containing 9 ml of sterile PBS. The biofilms were removed and dispersed by 30 s of vortexing, 2 min of sonication, followed by an additional 30 s of vortexing. Serial tenfold dilutions of the resulting bacterial suspension were prepared using sterile PBS, and the dilutions were plated on Tryptic Soy Agar. The plates were then incubated for 24–48 h at 37 °C. After incubation, the colonies on the plates were counted and the number of colony-forming units (CFU) for each disk was calculated.

The CFUs for each disk were log_10_-transformed. Log differences were calculated for each textured material relative to the untextured (smooth) material in each experiment. Statistical analysis was performed on the log_10_(CFU/disk) values. A mixed-effects analysis of variance (ANOVA) was fit to the log differences for each time point (2 or 24 h) separately using Minitab^®^ 18 software. The ANOVA included experiment as the random effect; and species, implant type and the two-way interaction as fixed effects. Because interaction plots and tests suggested an interaction between species and implant type, the log differences for each species and time point were analyzed separately to investigate the interaction. Pair-wise comparisons were conducted using Tukey’s multiple comparison procedure.

### Surface Area Adjustment

To convert the CFU/disk data to CFU/cm^2^, the following formula was used:

$${\text{CFU}}/{\text{cm}}^{2} = \left( {{\text{CFU}}/{\text{disc}}} \right) \, \times \, 1/{\text{disc}}\;{\text{area }}\left( {{\text{cm}}^{2} } \right) \, \times \, 1/{\text{surface}}\;{\text{area }}\left( {{\text{mm}}^{2} } \right) \, \times \, 100 \, \left( {{\text{mm}}^{2} /{\text{cm}}^{2} } \right).$$

These CFU/cm^2^ data were log_10_-transformed, then normalized and analyzed as described above.

### Confocal Scanning Laser Microscopy

Implant samples were stained with the nucleic acid stain SYBR Green^®^ (Life Technologies) to image bacteria and examined using a Leica SP5 confocal scanning laser microscope. Image processing was done using Imaris™ software.

## Results

### Surface Metrology

**Fig. 1:**
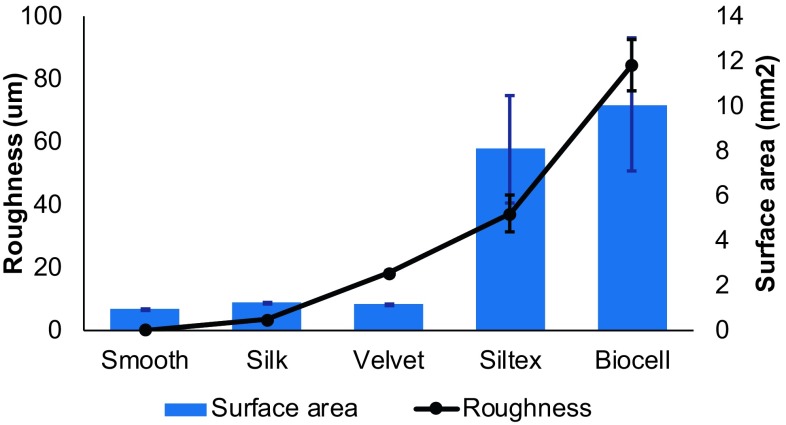
Surface metrology results for the breast implant surfaces evaluated in this study. The two least rough surfaces (Silk and Velvet) had similar surface areas as did the two most rough surfaces (Siltex and Biocell)

Surface area and roughness measurement results are summarized in Fig. 1. Surface area increased with roughness. The surface area was similar among the three least rough surfaces (Smooth, Silk, and Velvet) and among the roughest surfaces (Siltex and Biocell).

### Bacterial Attachment and Biofilm Growth

**Fig. 2:**
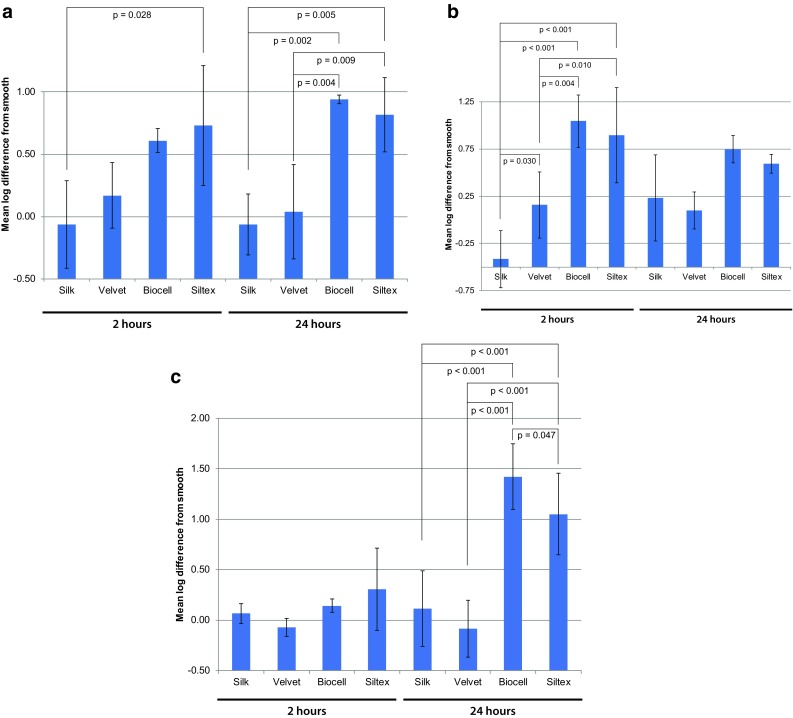
Summary log difference from smooth data for *S. epidermidis* (**a**), *P. aeruginosa* (**b**), and *R. pickettii* (**c**). Error bars indicate ± standard deviation from the mean. Positive values indicate more attachment/biofilm formation than Smooth, while negative values indicate less attachment/biofilm formation than Smooth. Overall, the Biocell and Siltex textures had greater differences from Smooth [i.e., more attached bacteria (2 h) and biofilm formation (24 h)] than the Silk and Velvet textures

Samples collected after 2 h of exposure represented bacterial attachment, as this time period was too short to allow for significant biofilm development on the surface. The samples collected after 24 h represented biofilm accumulation due to both attachment and growth. Various parameters (e.g., inoculum, ambient conditions, and medium batch) can influence variability between repeat experiments. These parameters should be equivalent for all the samples within each experiment. Thus, it is useful to express the results relative to an internal control within each experiment. In this research, the amount of biofilm on each textured material was expressed relative to the untextured (Smooth) material for each experiment (as a log difference from Smooth). The log differences from Smooth for each texture, species, and time point are shown in Fig. 2. Figure 2a is for *S. epidermidis,* Fig. 2b is for *P. aeruginosa, and* Fig. 2c is for *R. pickettii.* Negative log differences for the Silk and Velvet reflected that these textures often had less bacterial attachment/growth than the Smooth surface, although these differences were not statistically significant (*p* > 0.05). Overall, considering all species and time points, the Biocell and Siltex textures had statistically significantly more bacterial attachment and biofilm formation than the Velvet or Silk textures (*p* < 0.001).

Figure 2 suggests that there were interactions between species and implant type, although Silk and Velvet always had less biofilm than Siltex or Biocell. Pair-wise comparisons of the four textures revealed differences between species and time points for each species. For *S. epidermidis* at 2 h, there was a statistically significant difference between Silk and Siltex (*p *= 0.028). At 24 h, Silk and Velvet had significantly less biofilm than Siltex (*p *= 0.005) and Biocell (*p *= 0.002). For *P. aeruginosa* at 2 h, the Silk texture had significantly less biofilm than Velvet (*p *= 0.030), Siltex (*p *< 0.001), and Biocell (*p *< 0.001), while the Velvet texture had significantly less biofilm than the Siltex (*p *= 0.010) and Biocell (*p *= 0.004) textures. There were no statistically significant differences between any of the textures for *P. aeruginosa* at 24 h (*p* > 0.05). For *R. pickettii* at 2 h, there were also no significant differences between any of the textures. However, at 24 h for *R. pickettii*, the Silk and Velvet textures had significantly less biofilm than Siltex (*p *< 0.001) and Biocell (*p *< 0.001) textures. The Siltex texture also had significantly less biofilm than the Biocell texture (*p *= 0.047). Thus, *P. aeruginosa* showed significance differences between textures for bacterial attachment (2 h) but not for biofilm formation (24 h). In contrast, there were no significant differences for attachment of *R. pickettii*, but there were significant differences between textures for biofilm formation. *S. epidermidis* was similar to *R. pickettii*, with only one significant difference for attachment (Silk vs. Siltex) and many significant differences between textures for biofilm formation.

The data were also analyzed after adjustment for the differences in surface area of the textures (Fig. 1). There were no statistically significant differences between the textures for all species and time points except for *P. aeruginosa* at 2 h and *R. pickettii* at 2 and 24 h. For *P. aeruginosa* at 2 h, the Silk texture had significantly less biofilm than the Velvet (*p *= 0.024) and Biocell textures (*p *= 0.035). For *R. pickettii* at 2 h, Biocell had significantly less biofilm than Silk (*p *= 0.008) and Velvet (*p *= 0.016), and Silk had significantly less biofilm than Siltex (*p *= 0.044). The differences from smooth for *R. pickettii* at 2 h were similar for all of the textures, so when adjusted for surface area the higher surface area textures (Siltex and Biocell) were reduced more than the textures with less surface area. For *R. pickettii* at 24 h, Silk and Velvet had significantly less biofilm than Biocell (*p* = 0.006).

### Confocal Scanning Laser Microscopy

**Fig. 3:**
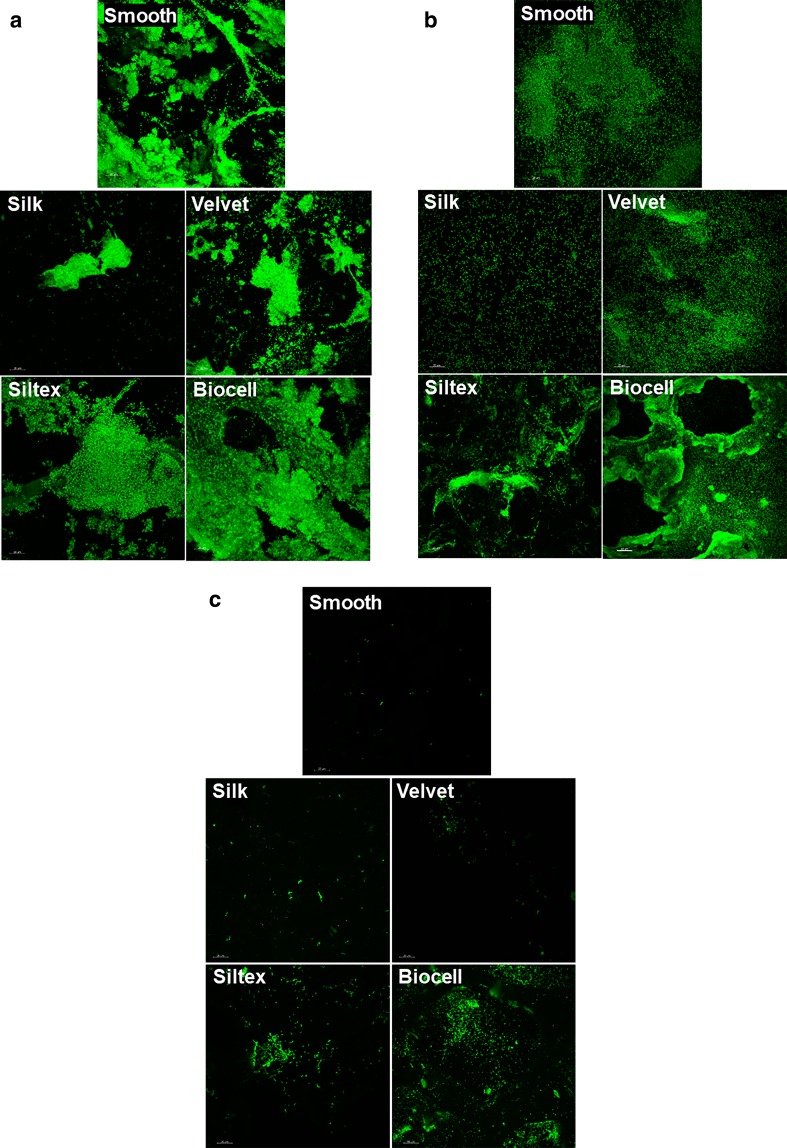
CSLM images of *S. epidermidis* (**a**), *P. aeruginosa* (**b**), and *R. pickettii* (**c**) biofilms after 24 h of growth on breast implant surfaces. For all three species, more biofilm was observed on the Biocell and Siltex textures than the Silk and Velvet textures

Overall, the CSLM results agreed with the plate count results, indicating there was more bacterial attachment and biofilm formation on the Biocell and Siltex textures than the Silk and Velvet textures. After 2 h of exposure, individual cells dispersed across the surfaces were observed (data not shown). After 24 h of exposure, there were more bacteria on the surfaces, with relatively thick *S. epidermidis* and *P. aeruginosa* biofilm accumulation on the Siltex and Biocell textures (see Figs. 3–5). *R. pickettii* formed less biofilm than *S. epidermidis* or *P. aeruginosa*, which also agreed with the plate count results.

## Discussion

Overall, there was more bacterial attachment and biofilm formation on the Biocell and Siltex textures than on the Silk and Velvet textures. This is related to the surface area of the implant materials. As shown in Fig. 1, the rougher Biocell and Siltex textures have more surface area. Thus, for the same size sample, these textures accumulate more biofilm than a smoother texture. When the viable plate count data were adjusted for surface area (CFU/cm^2^ rather than CFU/disk), there were no significant differences between the textures when data were pooled for all species and time points.

Although there was a clear correlation between biofilm accumulation and surface area in this study, other surface roughness parameters, such as skewedness and kurtosis, may also impact biofilm accumulation. In statistics, skewedness refers to the asymmetry of the probability distribution of a variable around the mean. For surface roughness, skewedness describes the peak heights and valley depths above or below the surface plane. Positive skewedness indicates more peaks than valleys on the surface compared to the mean height, while negative skewedness means fewer peaks than valleys. In statistics, kurtosis is a measure of the broadness of the tails in a probability distribution, or how wide or sharp is the peak and is often compared to the normal distribution. For surface roughness, it is a measure of the wideness or sharpness of the peaks. Overall, these parameters describe the regularity of the surface texture. More simply, we can view the surface texture as regular or not. In animal studies performed by our group (unpublished data), there is lower tissue integration with high kurtosis (high uniformity of the surface) and higher tissue integration with low kurtosis (lower uniformity of the surface). This may correlate with low bacterial adhesion in high kurtosis and higher bacterial adhesion in low kurtosis.

The etiologies of both CC and BI-ALCL are likely multifactorial. Indeed, textured implants have been shown to have lower rates of CC than smooth implants for submuscular placement [9, 10], even though they would be expected to harbor more biofilm. In the case of BI-ALCL, chronic inflammation has been suggested as a mechanism for disease development. In addition to biofilms [6, 8], this chronic inflammation could be the result of the toxicity of silicone or silicone breakdown products [11] as well as the direct effects of implant surface characteristics such as hydrophobicity and texture [12]. Certainly patient-specific factors, such as JAK1 and STAT3 mutations in BI-ALCL [13], likely also play a role.

Despite the overall trends, there were differences between species. For *S. epidermidis* at 2 h, the only statistically significant difference was between the Silk and Siltex textures, while at 24 h there were significant differences between the Silk and Velvet textures and the Biocell and Siltex textures. A previous in vitro study also compared *S. epidermidis* biofilm formation on smooth and textured breast implants at various time points, although the type of texture was not specified [14]. The log differences in that study (approximately 1 log at 2 h and 2 logs at 24 h) were slightly higher at two hours and much higher at 24 h than the current study. This was likely due to the different biofilm growth conditions used. CSLM results from that study were similar to the current study, with thick biofilms forming on the textured implant and relatively thin biofilms developing on the smooth implant. In the current study, similar results to *S. aureus* were seen for *R. pickettii*, where there were no statistically significant differences at 2 h and differences between the textures at 24 h. In contrast, for *P. aeruginosa* there were statistically significant differences between the textures at 2 h but not at 24 h. These results demonstrate that multiple species and time points should be used to assess biofilm formation on biomaterials to avoid misleading results.

Although this in vitro study demonstrated more biofilm accumulation on the rougher textures, in vivo conditions are more complex. In particular, the immune response to the implant and associated bacteria/biofilm likely plays an important role. For example, recent in vitro studies have shown that highly textured implant surfaces induce higher levels of proinflammatory gene expression and cytokine production by macrophages and monocytes than smoother surfaces [12, 15]. In vitro systems including both biofilm and leukocytes, along with in vivo investigations, may help to elucidate the complex interactions between bacteria and the immune response on implant surfaces.

## Conclusion

Overall, the results of this study indicate that rougher breast implant textures with greater surface areas (Siltex and Biocell) accumulate more biofilm than smoother textures with less surface area (Silk and Velvet). However, there were differences between species and time points, with *P. aeruginosa* attaching more to the textures with greater surface area but not developing more biofilm on these textures, whereas *S. epidermidis* and *R. pickettii* displayed more difference between textures for biofilm formation.
